# American lobster postlarvae alter gene regulation in response to ocean warming and acidification

**DOI:** 10.1002/ece3.7083

**Published:** 2020-12-29

**Authors:** Maura Niemisto, David M. Fields, K. Fraser Clark, Jesica D. Waller, Spencer J. Greenwood, Richard A. Wahle

**Affiliations:** ^1^ Darling Marine Center University of Maine School of Marine Sciences Walpole ME USA; ^2^ Bigelow Laboratory for Ocean Sciences East Boothbay ME USA; ^3^ Department of Animal Science and Aquaculture Faculty of Agriculture Dalhousie University Bible Hill NS Canada; ^4^ Maine Department of Marine Resources West Boothbay Harbor ME USA; ^5^ Department of Biomedical Sciences Atlantic Veterinary College University of Prince Edward Island Charlottetown PEI Canada

**Keywords:** crustaceans, gene expression, *Homarus americanus*, joint stressors, larvae, ocean acidification, ocean warming, RNA‐seq

## Abstract

Anthropogenic carbon emissions released into the atmosphere is driving rapid, concurrent increases in temperature and acidity across the world's oceans. Disentangling the interactive effects of warming and acidification on vulnerable life stages is important to our understanding of responses of marine species to climate change. This study evaluates the interactive effects of these stressors on the acute response of gene expression of postlarval American lobster (*Homarus americanus*), a species whose geographic range is warming and acidifying faster than most of the world's oceans. In the context of our experiment, we found two especially noteworthy results: First, although physiological end points have consistently been shown to be more responsive to warming in similar experimental designs, our study found gene regulation to be considerably more responsive to elevated *p*CO_2_. Furthermore, the combined effect of both stressors on gene regulation was significantly greater than either stressor alone. Using a full factorial experimental design, lobsters were raised in control and elevated *p*CO_2_ concentrations (400 ppm and 1,200 ppm) and temperatures (16°C and 19°C). A transcriptome was assembled from an identified 414,517 unique transcripts. Overall, 1,108 transcripts were differentially expressed across treatments, several of which were related to stress response and shell formation. When temperature alone was elevated (19°C), larvae downregulated genes related to cuticle development; when *p*CO_2_ alone was elevated (1,200 ppm), larvae upregulated chitinase as well as genes related to stress response and immune function. The joint effects of end‐century stressors (19°C, 1,200 ppm) resulted in the upregulation of those same genes, as well as cellulase, the downregulation of calcified cuticle proteins, and a greater upregulation of genes related to immune response and function. These results indicate that changes in gene expression in larval lobster provide a mechanism to respond to stressors resulting from a rapidly changing environment.

## Graphical abstract

Using nextgen transcriptomic sequencing methodologies, our study evaluates the single and joint effects of elevated temperature and CO_2_ on gene regulation in the postlarval stage of the American lobster. Our study shows gene regulatory response to be considerably more affected by elevated *p*CO_2_ levels than elevated temperature, contrasting what has been shown in studies of physiological end points of larval crustaceans, as well as a strong joint effect of simultaneous stressors.

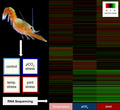

## 1 INTRODUCTION

The release of anthropogenic carbon emissions into the earth's atmosphere has resulted in large‐scale changes in oceanic temperature and pH that have an impact on marine ecosystems and fisheries (IPCC, 2019). IPCC scenarios project *p*CO_2_‐atm will continue to climb resulting in an average temperature increase of 0.6–2.0°C and a pH drop of 0.06–0.32 units, by the end of the century (IPCC, 2013). These rapid, concurrent stressors are causing concern for the survival of some marine organisms and resilience of key fisheries (Gledhill et al., 2015).

The impacts of ocean acidification (OA) and ocean warming (OW), as single and joint stressors on marine organisms, are complex and species‐specific (Fabry et al., 2008; Kroeker et al., 2013; Kurihara, 2008; Whitman & Pörtner, 2013). As genomic analysis techniques increase in efficiency, transcriptomic analyses have become important metrics for quantifying the expression of stress‐related genes (Evans & Hofmann, 2012), and enable the examination of a broad range of genetic responses to environmental change on organisms (Harms et al., 2014; Todgham & Hofmann, 2009). Not only can these techniques increase our understanding of the scope of the organismal response, but they have the potential to detect molecular compensation for environmental stress that may otherwise go undetected using more traditional physiological studies (Gracey, 2007).

The American lobster (*Homarus americanus*) is the single most valuable fishery in North America and has particular socio‐economic importance in the Gulf of Maine (National Marine Fisheries Service, 2020). It also has a long history as a well‐studied organism in physiology, development, and ecology (Factor, 1995). The American lobster's range extends from Northeastern Canada to the mid‐Atlantic states of the United States, where sea surface temperatures during their larval seasons range from below 12°C in the north to over 20°C at the southern extent of its range and in shallow regions (Oulette et al., 2003; Quinn et al., 2013). The entirety of this area falls within the Northwest Atlantic and Gulf of Maine (GoM), where sea surface temperature is warming at a faster rate than the majority of the world's ocean (0.026°C per year since 1980) with a predicted increase ranging 0.03–0.05°C per year (Balch et al., 2012; LeBris et al., 2017; Pershing et al., 2015; Thomas et al., 2017). The region is also particularly susceptible to higher rates of acidification, due to depressed buffering capacity from freshwater inputs of rivers and incoming, relatively fresh currents, resulting in a low aragonite saturation level (Fabry et al., 2008; Gledhill et al., 2015; Salisbury et al., 2008). These conditions place urgent concern and interest in how this species will respond to climate‐related stressors, particularly for pelagic life stages in the upper water column. The center of the American lobster fishery has already demonstrated a northward range shift in response to the rapidly warming temperatures within this region, and there is some concern this pattern may continue, affecting fisheries to the south (LeBris et al., 2018).

Here, we evaluate the joint effects of elevated *p*CO_2_ and sea surface warming associated with end‐century projected oceanic conditions using next‐generation RNA sequencing (RNA‐seq) to monitor gene expression changes in early life stage American lobster (*Homarus americanus*). Postlarval *H. americanus* have demonstrated a shift in gene regulatory response under increasing temperature treatments representative of end‐century SST warming (Harrington et al., 2020). However, to our knowledge, this is the first report of the impact of changes in *p*CO_2_ and temperature, as joint stressors, on gene expression in the early life stages *Homarus americanus*, and one of the few examining these two stressors on planktonic decapods as a group (Walther et al., 2010, 2011; Harms et al., 2014; Small et al., 2015; Waller et al., 2017).

Surprisingly, little is known about how this species will respond to end‐century *p*CO_2_ and temperatures as concurrent stressors, but it is suggested that molting through several key developmental pelagic and benthic life stages in a single season makes this species especially vulnerable to these oceanic changes (Gledhill et al., 2015; Kurihara et al., 2007; Pörtner & Farrell, 2008; Waller et al., 2017). Physiology and metabolic demand differ between life stages, indicating stage‐specific vulnerability and developmental success are closely tied to environmental conditions; as a result, few individuals survive these biological bottlenecks to settlement stage (Hines et al., 2014; Small et al., 2015; Waller et al., 2017). Thus, the final pelagic stage of this organism, the postlarvae, serves as an ideal and relevant organism for this study, since it must complete all larval ontogenetic stages within the water column to reach the stage of recruitment.

Results from physiological studies on larval and early juvenile *Homarus* congeners suggest a range of responses to end‐century acidification and warming as joint stressors (Agnalt et al., 2013; Keppel et al., 2012; Menu‐Courey et al., 2019; Rato et al., 2017; Ries et al., 2009; Small et al., 2015; Waller et al., 2017). Elevated temperature and *p*CO_2_ can interact to cause changes in behavior, carapace length, carbon content, and development time; however, reports are conflicting in some cases (Small et al., 2015; Waller et al., 2017). The few studies to date that have tested thermal and *p*CO_2_ effects together under similar experimental designs suggest that larval or postlarval physiological and behavioral response to predicted end‐century warming may be greater than that of *p*CO_2_ alone (Agnalt et al., 2013; Small et al., 2015; Waller et al., 2017).

The primary goal of this study was to examine the gene regulatory response of the postlarval *Homarus americanus* to end‐century projected sea surface temperature and *p*CO_2_ both as independent and joint stressors, and to better understand organismal response on a molecular level to predicted OW and OA. Based on previous studies using a similar experimental design (Small et al., 2015; Waller et al., 2017), we hypothesized that the gene regulatory response to temperature would be more pronounced than to increased *p*CO_2_ which is consistent with the prior physiological observations of larvae and postlarvae noted above. Utilizing next‐generation sequencing, our results suggest strong direct responses to *p*CO_2_ and greater interactive effects of *p*CO_2_ and temperature on genes associated with immune functions and shell formation.

## 2 METHODS

### 2.1 Experimental design

The Rhode Island Department of Environmental Management's Fisheries Section collected seven ovigerous female American lobsters (*Homarus americanus*) from the coastal waters of Rhode Island in summer 2016. Lobsters were transported to the University of Maine's Darling Marine Center, Walpole, ME, and held in aerated, 300 L hatching tanks at ~15°C until hatching. Upon hatching (±6 hr), stage I larvae were transported to Bigelow Laboratory for Ocean Sciences and distributed randomly in 18 × 20 L buckets, pre‐equilibrated to the experimental treatments. Each bucket was stocked with 250 larvae, resulting in a starting average density of 12.5 larvae/L. Larvae were fed live newly hatched *Artemia salina* daily, in excess, until they reached postlarval stage (15–31 days).

The experiment was designed as a full factorial with two temperatures and two levels of *p*CO_2_, representing four distinct treatment groups: control, elevated temperature, elevated *p*CO_2_, and elevated temperature and *p*CO_2_. Temperatures were replicated from previous larval studies on *H. americanus* (Waller et al., 2017). All tanks were held in a temperature‐controlled room at 16°C (±0.1°C), representative of the average summer sea surface temperature during larval season in Midcoast Maine (Mackenzie, 1988; Quinn & Rochette, 2015). The elevated temperature treatment (19°C; ±0.5°C) was achieved using Hydor submersible aquarium heaters, representing an end‐century increase of 3°C (IPCC, 2013). This temperature (19°C) is also just below the thermal threshold recorded for lobsters in the region, where the mortality rate increases with temperature (Mackenzie, 1988).

The two *p*CO_2_ treatments (400 ppm and 1,200 ppm) were created by mixing pure CO_2_ with CO_2_‐stripped, compressed air to create predetermined concentrations of gasses (Waller et al., 2017). The two *p*CO_2_ concentrations represented the ambient atmospheric concentration (400 ppm), and an elevated *p*CO_2_ concentration to generate pH values consistent with end‐century projected estuarine and coastal regions (~7.6) (Gledhill et al., 2015; IPCC, 2013; Table 1). Each of the four treatment combinations was maintained in triplicate for a total of 12 tanks.

**TABLE 1:** Water Chemistry parameters during the course of experiment. All parameters list average value and *SD* through experimental period

Treatment	Temperature (°C)	Salinity (ppt)	pH	ΩCa	ΩAr
400 ppm 16°C	16.6 ± 0.5	30.3 ± 0.8	7.94 ± 0.06	2.34 ± 0.10	1.45 ± 0.06
1,200 ppm 16°C	17.0 ± 0.4	30.0 ± 0.8	7.56 ± 0.01	1.16 ± 0.025	0.72 ± 0.02
400 ppm 19°C	18.7 ± 0.4	30.2 ± 0.9	7.89 ± 0.03	2.44 ± 0.25	1.52 ± 0.16
1,200 ppm 19°C	19.5 ± 1.0	29.9 ± 0.7	7.63 ± 0.01	1.42 ± 0.004	0.88 ± 0.002

Abbreviations: ppt, parts per thousand; *SD*, Standard Deviation; ΩAr, aragonite saturation; ΩCa, calcite saturation.

Salinity, temperature, and pH were monitored daily. Salinity was measured using an Oakton SALT meter, and pH and temperature were monitored using a Thermo Orion 3‐star benchtop pH probe, calibrated using NIST buffers. To calculate the carbonate chemistry of the water, total pH (pH_t_) was measured spectrophotometrically (Hitachi U‐310 dual‐beam, Hitachi, USA) using the pH‐sensitive indicator dye m‐cresol purple (Sigma‐Aldrich) following SOP (standard operating procedure 6b: Dickson et al., 2007; Table 1). Total alkalinity (ALK_t_) was measured from samples preserved in mercuric chloride using a Metrohm 888 Titrando (Metrohm, USA). Both pH_t_ and ALK_t_ were measured twice a week throughout the experiment and were used to calculate carbonate chemistry parameters (*p*CO_2_, [HCO_3_^−^], [CO_3_^2−^], Ω_Ar_, Ω_Ca_) using the CO2SYS2.1 system (Lewis et al., 1998).

Communal rearing tanks were maintained under treatment conditions for the entirety of larval development. Upon reaching stage IV, we separated postlarvae into individual containers for 48 hr, maintaining treatment conditions in each. After this period, postlarvae were starved for 24 hr to remove residual *Artemia salina* genetic material from their digestive tract. Postlarvae were rinsed in UV‐sterilized 0.2 µm filtered seawater and placed in sterile cryotubes with 3 ml RNA*later* (Ambion, USA). All samples were flash frozen and stored at −80°C.

### 2.2 RNA extraction

RNA was extracted following the method of Clark, Acorn, et al. (2013) and RNA‐seq performed on a total of 11 animals drawn from the four treatment combinations (*n* = 3 in all treatments except the control 16°C, 400 ppm treatment, where *n* = 2). Briefly, the preserved postlarvae were individually homogenized in 1 ml Trizol (TH electric homogenizer; OMNI International), placed in a chloroform/Trizol mixture (200 µl chloroform/ml of Trizol), and incubated for 3 min at room temperature. Samples were then centrifuged at 12,000 *g* at 4°C (15 min), and the collected supernatant was added to an equal volume of 100% ethanol. RNA was extracted with a RNeasy kit (Qiagen) with an on‐column DNasel digestion, and quantified using a NanoDrop1000 spectrophotometer (Thermo Fisher Scientific). Quality was verified with the Agilent Bioanalyzer 2100 and RNA Nano 6000 chips.

### 2.3 Bioinformatics

The collected RNA was sequenced at Genome Quebec (Montreal, PQ, Quebec) with libraries prepared using a TruSeq Stranded Total RNA Prep kit (Illumina). Quality was assessed and PE100 sequencing was performed on an Illumina HiSeq4000 using all samples on a single lane. Raw sequence reads were uploaded onto the main Galaxy web platform and analyzed on the public server at usegalaxy.org (Afgan et al., 2016). We assessed quality of raw reads with FastQC (Andrews, n.d.; Galaxy Version 1.0.0) and trimmed adapter sequences using Trim Galore! (Krueger, n.d.; Galaxy Version 0.4.3.1). A de novo transcriptome was constructed using Trinity (Langmead et al., 2009, Galaxy Version 0.0.1). Trimmed sequences were mapped to the transcriptome using HISAT2 (Daehwan et al., 2015; Galaxy Version 2.1.0), and a count file for each sample was generated using StringTie (Pertea et al., 2015; Galaxy Version 1.3.4). Count files were merged using StringTie Merge (Pertea et al., 2015; *Galaxy* Version 1.3.4.) and normalized using featureCounts (Liao et al., 2013; Galaxy Version 1.6.3.).

Differential expression of transcripts was analyzed statistically between treatments using both DESeq2 (Love et al., 2014; Galaxy Version 2.11.40.2) and edgeR (Conesa et al., 2016; Liu et al., 2015; Robinson et al., 2009; Galaxy Version 3.20.7.2), as the integration of multiple methods has been shown to improve accuracy and reduce error rates (Costa‐Silva et al., 2017). While edgeR and DESeq2 are based on a negative binomial distribution and are both recommended for small sample sizes, DESeq2 uses a geometric normalization method, whereas edgeR calculates a weighted mean of log‐ratios for normalization (Dillies et al., 2013; Nguyen et al., 2018). The integration of both tools in our methodology produces a more robust gene expression analysis (Nguyen et al., 2018).

RNA transcripts were compared from *H. americanus* postlarvae raised in the control treatment (400 ppm, 16°C) to the other temperature/*p*CO_2_ treatment concentrations. Results were depicted graphically as venn diagrams to show the proportion of transcripts related to each treatment (Heberle et al., 2015), and as “volcano plots” to show direction, magnitude, and statistical significance of the differential expression of each transcript under each experimental treatment relative to the control. Differentially expressed transcripts were uploaded onto Blast2GO (Götz et al., 2008) and assigned gene names using Blastx (Camacho et al., 2008) against the GenBank protein nr database, functional terms were assigned with Gene Ontology (Ashburner et al., 2000; Gene Ontology Consortium, 2019), enzyme numbers were assigned with KEGG (Kanehisa, 2019; Kanehisa & Goto, 2000; Kanehisa et al., 2019) and domain information assigned with Interpro (Mitchell et al., 2019; Blast2GO 4.0.2).

Several genes of interest related to carapace formation were selected for more in‐depth investigation based on their functional importance and their response to elevated *p*CO_2_. Specific genes families (and the number of genes) related to calcification and shell formation were tracked through all treatments: cuticle proteins (13), cuticle protein binding molecules (CBM) (2), chitin‐binding protein (3), chitinase (4), calcification‐associated peptides (2), and arthrodial cuticle proteins (4). In addition, we examined the differential expression of several transcripts related to immune response and function (Clark & Greenwood, 2016) including heat shock proteins (HSP) (3), hemocyanin subunits (27), mannose‐binding proteins (5), crustin (2), c‐type lectin (2), glutathione S‐transferase (2), and octopamine receptor (1), as indicators of a stress response.

## 3 RESULTS

**FIGURE 1:**
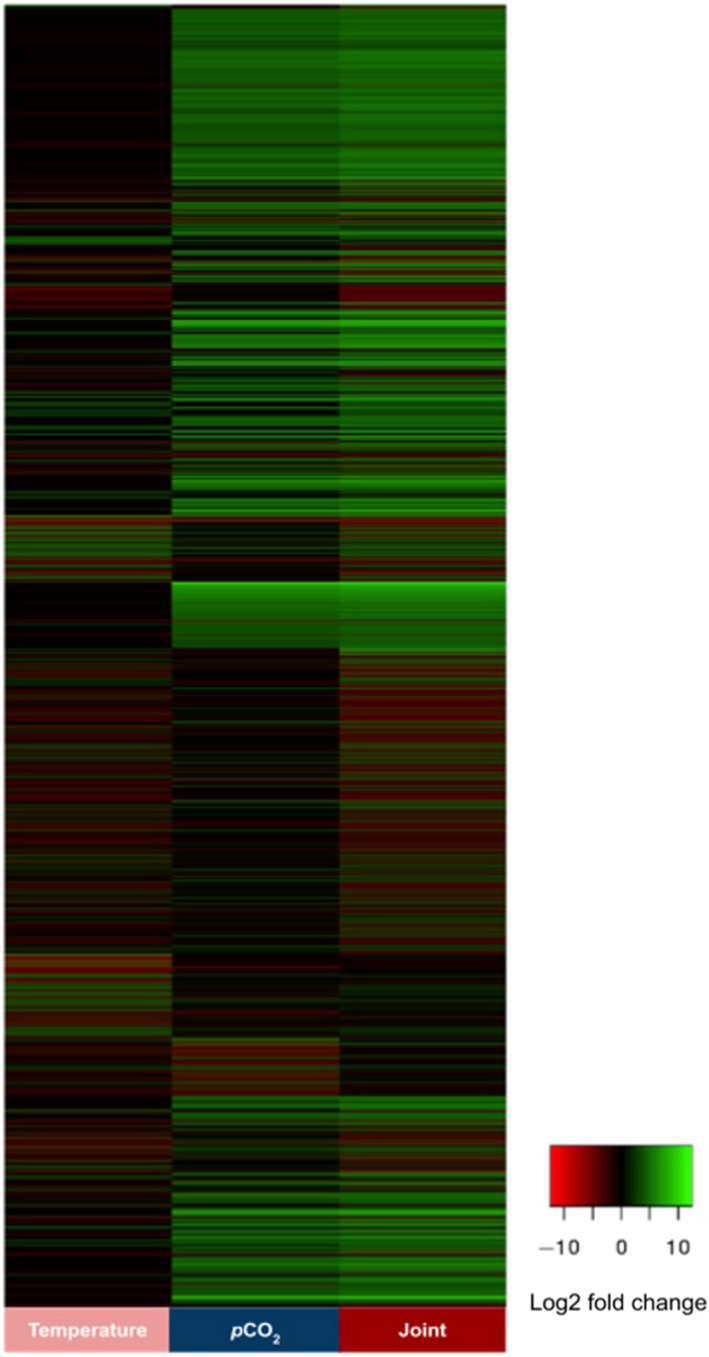
Heat map depicting expression of the 1,108 differentially expressed genes subject to single and joint stressor treatments, relative to control treatment of 400 ppm, 16°C. This represents only 0.27% of all transcripts identified. Colors represent absolute value of log2 fold change. Red represents a downregulation relative to the control, whereas green represents upregulation. More than half of these genes (55%) were functionally annotated and subject to further analysis (Figures 2, 3, 4, 5, 6)

A de novo assembled transcriptome was generated containing 414,517 unique transcripts. The minimum transcript length was 201 bp, and the maximum was 21,784 bp. The N50 transcript length was 1,909. Using the DESeq2, we found 1,108 transcripts (0.27% of all transcripts) that were differentially expressed (upregulated or downregulated) across treatments (Figure 1). The vast majority of all identified transcripts (99.73%) were therefore not differentially expressed.

**FIGURE 2:**
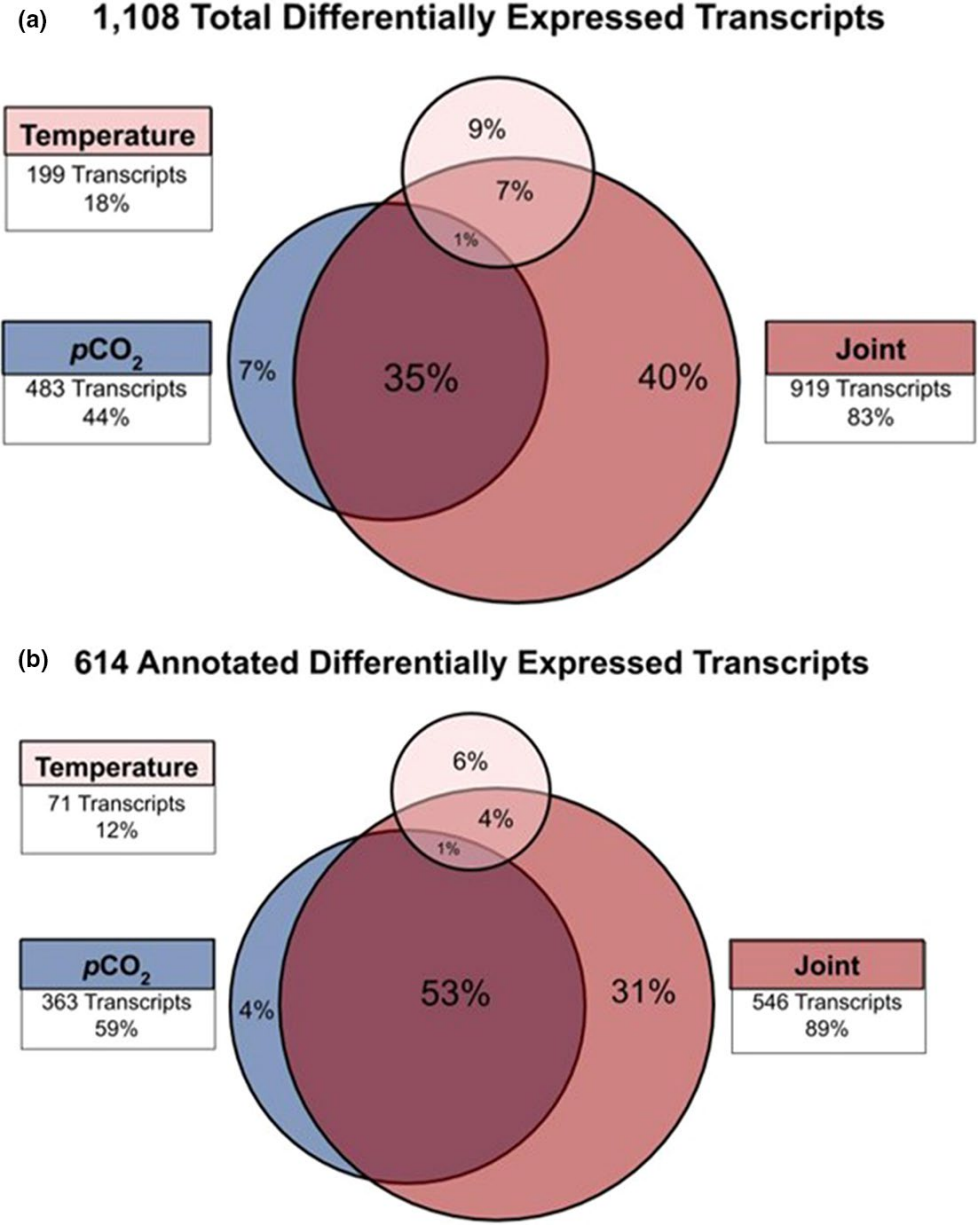
Percent of total (a) and annotated (b) differentially expressed transcripts as quantified by DESeq2 analysis within elevated temperature treatment (400 ppm 19°C), elevated *p*CO_2_ treatment (1,200 ppm 16°C), and joint temperature and *p*CO_2_ treatment (1,200 ppm 19°C). All differential expression is relative to control treatment (400 ppm 16°C)

Elevated temperature and *p*CO_2_ together had a stronger effect on gene expression than did either factor alone. Out of the 1,108 differentially expressed transcripts, elevated temperature alone induced the differential expression of 199 transcripts (18% of all differentially expressed transcripts) relative to the control conditions; elevated *p*CO_2_ alone induced differential expression of 483 transcripts (44%), and 919 transcripts (83%) were differentially expressed when both stressors were present (Figure 2a).

**FIGURE 3:**
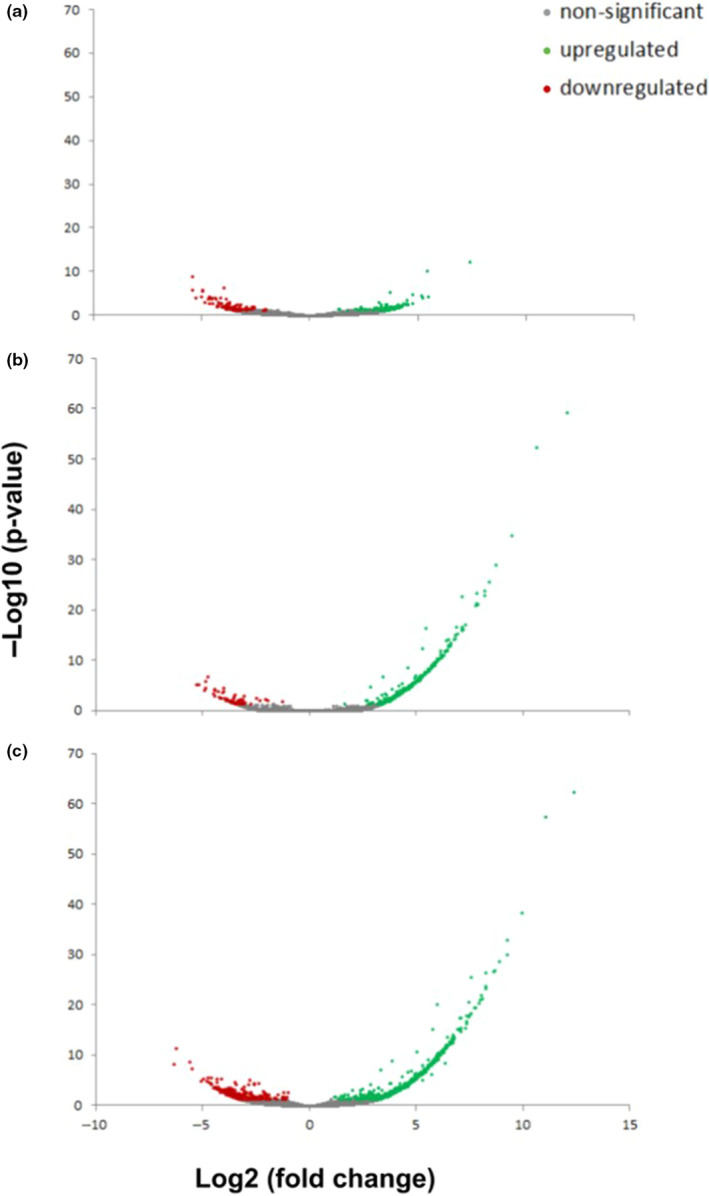
Volcano plots from DESeq2 analysis depicting the statistical significance (−Log10(*p*‐values)) of transcripts against the Log2 transformed magnitude of change of each transcript across samples of treatments of elevated temperature (a), elevated *p*CO_2_ (b), and elevated temperature and *p*CO_2_ (c), relative to the control condition. Values in green represent statistically significant upregulated and red represent downregulated genes in treatment samples relative to control treatment

Functional annotation was possible for 55% of the differentially expressed transcripts using Blast2GO (Figure 2b). As with the unannotated genes, we found the majority of differentially expressed annotated genes in the joint treatment of elevated temperature and elevated *p*CO_2_ (89% of annotated, differentially expressed transcripts) compared to only 59% in the treatment with only elevated *p*CO_2_ and 12% with only elevated temperature (Figure 2b). Overall, differentially expressed transcripts were predominantly upregulated in the elevated *p*CO_2_ treatment (1,200 ppm 16°C) and joint stressor treatment (1,200 ppm 19°C; Figure 3).

**FIGURE 4:**
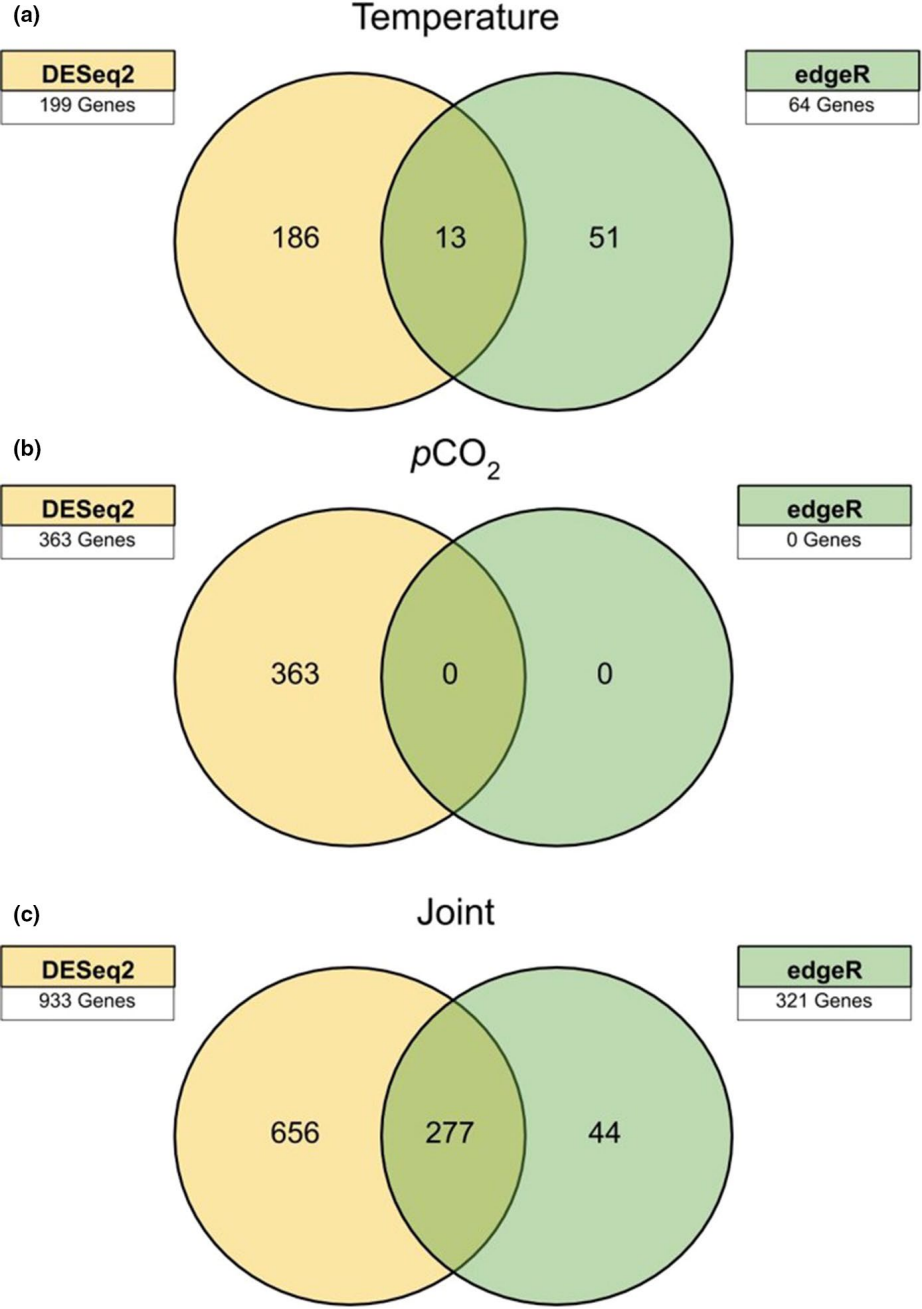
Comparison of number of annotated, statistically significant (*p* < .05), differentially expressed transcripts identified by DESeq2 and edgeR analysis within elevated temperature (a), elevated *p*CO_2_ (b), and joint elevated temperature and *p*CO_2_ treatments (c)

Although edgeR resulted in a more conservative number of statistically significant transcripts, 75.3% of those significantly differentially regulated transcripts were also identified with DESeq2. We found 7% of the differentially expressed DESeq2‐identified transcripts to be shared between both analyses for the elevated temperature treatment (Figure 4a). There were no differentially expressed transcripts within the *p*CO_2_ treatment using edgeR (Figure 4b). Conversely, there were 30% of the differentially expressed DESeq2 identified transcripts shared with edgeR within the joint stressor treatment, representing 71.9% of all identified differentially regulated transcripts via edgeR analysis (Figure 4c).

**FIGURE 5:**
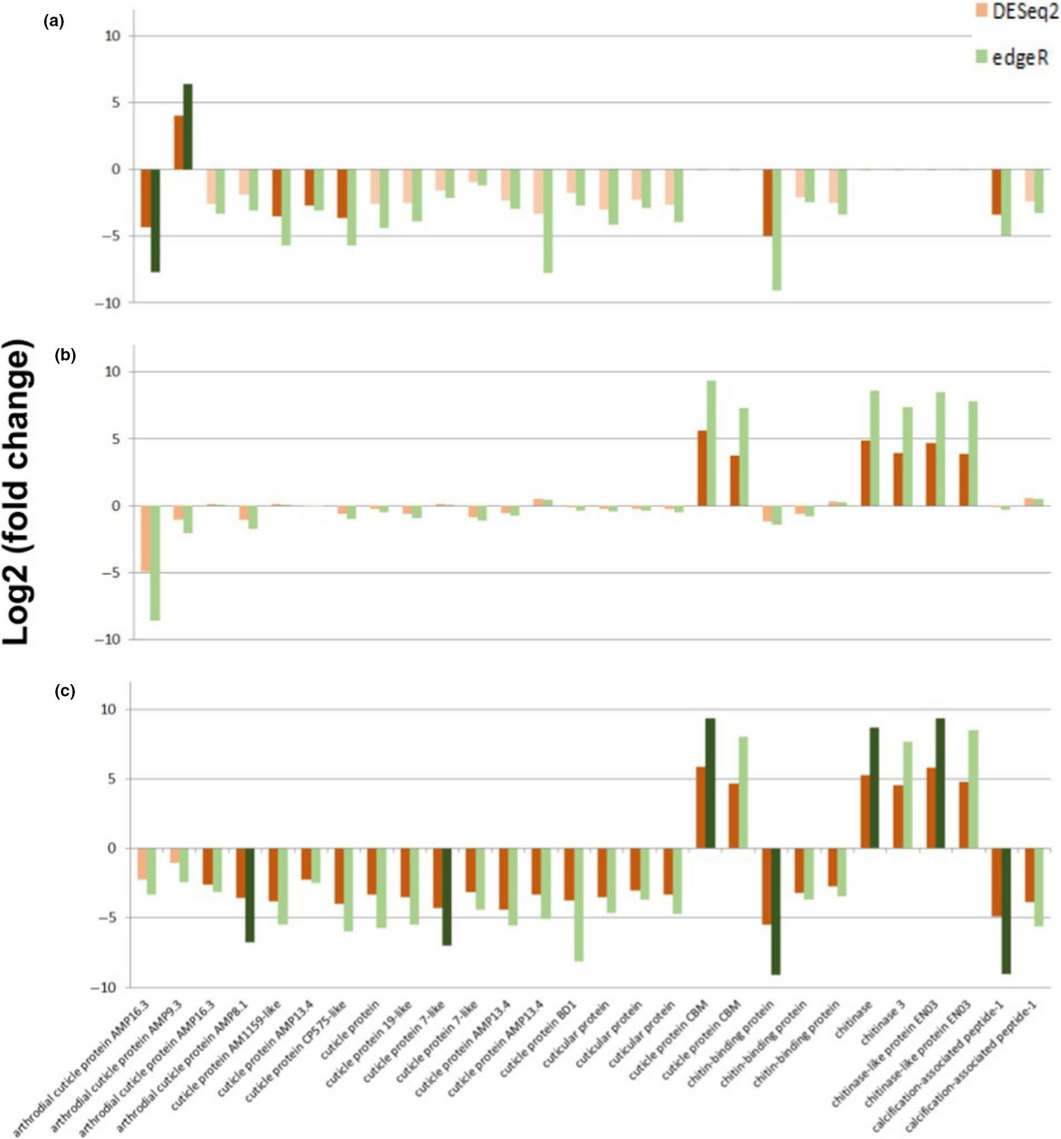
Response of lobster postlarval exoskeleton formation transcripts to elevated temperature and *p*CO_2_ by DESeq2 (orange) and edgeR (green) analyses. Depicted are Log2 fold change of 28 genes of interest at elevated temperature (a), elevated *p*CO_2_ (b), joint elevated temperature & *p*CO_2_ and (c) relative to gene expression under control conditions. Darkened colors indicate statistically significant outcomes (*p* < .05)

**FIGURE 6:**
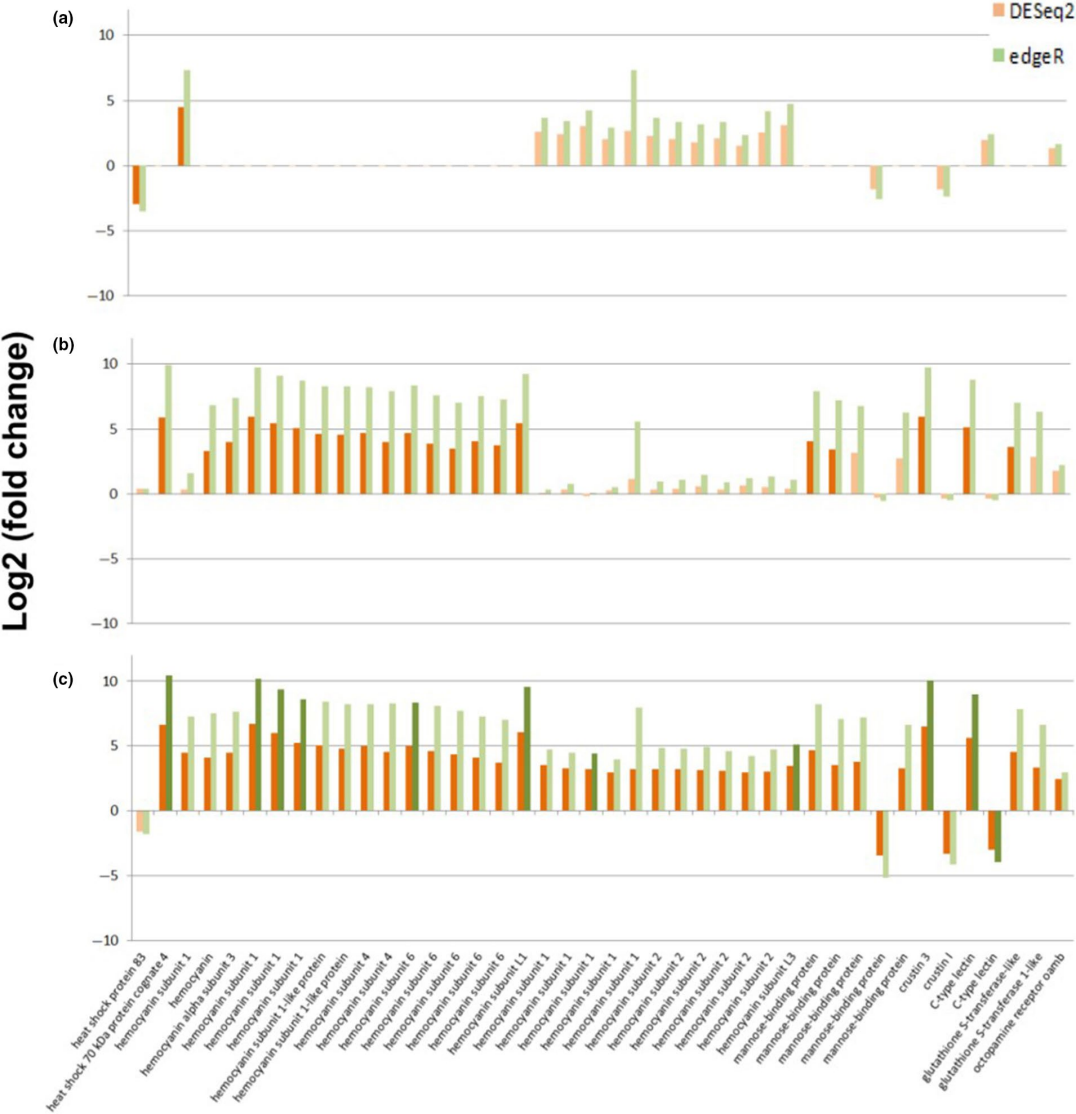
Response of lobster postlarval immune function transcripts to elevated temperature and *p*CO_2_ by DESeq2 (orange) and edgeR (green) analyses. Depicted are Log2 fold change of 42 transcripts of interest at elevated temperature (a), elevated *p*CO_2_ (b), joint elevated temperature and *p*CO_2_ treatment and (c) relative to gene expression under control conditions. Darkened colors indicate statistically significant outcomes (*p* < .05)

Analysis of annotated genes revealed that DESeq2 and edgeR were in 100% agreement with regard to the direction of gene regulation. While DESeq2 consistently found lower differential expression, more of these transcripts were found to be statistically significant (*p* < .05) using this analysis (Figures 5 and 6).

Genes involved in exoskeleton formation (cuticle proteins, chitin‐binding proteins, and calcification peptides) were largely downregulated in response to OW and OA, with few exceptions (Figure 5). In treatments where only temperature was elevated relative to control conditions, six of the 28 genes showed significant downregulation and one showed significant upregulation by at least one of the statistical analysis methods (Figure 5a). In treatments where only *p*CO_2_ was elevated, DESeq2 showed significant upregulation for cuticle protein binding molecules, chitinase, and chitinase‐like proteins, but edgeR resulted in no differential regulation for any of these genes (Figure 5b). In treatments where both temperature and *p*CO_2_ were jointly elevated, both methods indicated significant downregulation of cuticle proteins, chitin‐binding proteins, and calcification‐associated peptides, and upregulation of cuticle protein binding molecules, chitinase, and chitinase‐like proteins (Figure 5c).

With respect to the differential expression of genes associated with the immune response, in the elevated temperature treatment, postlarvae downregulated HSP83, and upregulated one transcript for hemocyanin subunit 1 according to the DESeq2 (Figure 6a). In the elevated *p*CO_2_ treatment, DESeq2 indicated significant upregulation of HSP70, 15 transcripts related to hemocyanin and its subunits, mannose‐binding units, crustin, C‐type lectin, and glutathione S‐transferase, but edgeR detected no statistically significant (*p* < .05) change in these transcripts (Figure 6b). In the joint stressor treatment, DESeq2 indicated postlarvae upregulated more transcripts related to hemocyanin and its subunits, octopamine receptor, in addition to the genes differentially regulated within the elevated *p*CO_2_ only treatment, whereas for edgeR, only 11 transcripts within this category were significantly differentially expressed (Figure 6c).

## 4 DISCUSSION

To our knowledge, this is the first study to examine how the joint stressors of these SST warming and acidification conditions may affect gene expression of American lobster postlarvae. DESeq2 analysis indicates that postlarval response to elevated *p*CO_2_ is more pronounced relative to warming than indicated in physiological and morphometric studies reported to date (Agnalt et al., 2013; Keppel et al., 2012; Menu‐Courey et al., 2019; Rato et al., 2017; Ries et al., 2009; Small et al., 2015; Waller et al., 2017). In addition, analysis of gene regulatory responses revealed an even greater response to joint effects of elevated *p*CO_2_ and temperature on transcripts involved in developmental processes and immune function of lobster postlarvae.

Contrary to the DESeq2 results, the analysis using EdgeR did not detect any statistically significant differential expression under the *p*CO_2_ treatment and detected fewer differentially expressed (DE) genes within other treatments. This may be due to the sample size in this study. While the accepted number of replicates in transcriptomic studies is not universally standardized, (Costa‐Silva et al., 2017; Stark et al., 2019), comparative analysis of different genetic methodologies certainly would benefit from greater sample numbers. That said, both methodologies are robust and well‐suited to small sample sizes, and have high accuracy rates (DESeq2 being one of the highest ranked methodologies compared to qRT‐PCR analyses) (Costa‐Silva et al., 2017).

Furthermore, direction of gene expression was in 100% agreement between the two methods, and the discrepancy in DE genes between methodologies lies in the normalization strategies of the tools (Dillies et al., 2013; Nguyen et al., 2018). The inclusion of multiple methods in our analysis reduces the likelihood of false negatives and provides greater confidence in the results when in agreement (Costa‐Silva et al., 2017).

The effects of elevated *p*CO_2_ and temperature on the transcription level associated with cuticle formation and calcification were treatment‐dependent. Elevated temperature resulted in downregulation of cuticle proteins and a calcification‐associated peptide. Calcification‐associated proteins act as acidic protein sites for nucleation of CaCO_3_ during larval biomineralization (Addadi & Weiner, 1985; Faircloth & Shafer, 2007). Downregulation indicates a temperature cost to calcification, though fewer genes were differentially regulated than in other treatments. Warming has been shown to reduce shell integrity in the mussel *Mytilus edulis* when food limited, an effect attributed to a reallocation of energy away from biomineralization in order to address temperature‐related increases in maintenance requirements (MacKenzie et al., 2014).

Under elevated *p*CO_2_, postlarval lobster upregulated chitinase and cuticle proteins associated with calcium binding, both of which are important components of exoskeleton development and remodeling. This result is consistent with previous findings in which larval lobster upregulate chitinase synthesis genes during ontogeny, presumably to support increased chitin synthesis and maintenance (Cohen, 2010; Hines et al., 2014). Juvenile lobsters also increase shell calcification under elevated *p*CO_2_ conditions (Ries et al., 2009; Whitely, 2011). The combination of end‐century temperature and *p*CO_2_ has an additive effect on exoskeleton forming genes causing additional cuticle protein downregulation. These findings mirror other studies that have examined skeletal formation in marine invertebrates when exposed to elevated temperature and acidity. Downregulation of calcification‐related genes was reported for pearl oysters (*Pinctada fucata*) after being exposed to end‐century conditions (Liu et al., 2012). Larvae of the purple sea urchin (*Strongylocentrotus purpuratus*), however, exhibit inhibited skeletal growth under elevated *p*CO_2_, but not at elevated temperatures (Padilla‐Gamino et al., 2013). Nevertheless, bivalve mollusks and urchins appear to have lower capacity to compensate for these elevated *p*CO_2_ than the relatively small set of crustaceans evaluated to date (Kurihara, 2008; Wood et al., 2008).

Heat shock proteins (HSP) were downregulated in lobster larvae under elevated temperature alone, but were upregulated when exposed to elevated *p*CO_2,_ and an even greater upregulation when *p*CO_2_ and temperature were elevated simultaneously. Heat shock proteins are molecular chaperones that are upregulated after exposure to stressful conditions to prevent improper folding or denaturation of proteins (Alberts et al., 2015; Flaherty et al., 1990; Kiang & Tsokos, 1998). Virtually, all organisms upregulate HSP expression as a method to alleviate physiologically stressful conditions (Evans & Hofmann, 2012). Thus, HSPs can modify an organism's thermal sensitivity and act as important biological stress markers (Tedeschi et al., 2015). When reared in elevated *p*CO_2_, or joint stressor conditions, HSP70 was the highest upregulated transcript in our immune or shell formation transcripts, suggesting a potentially prominent role in compensating for environmentally stressful conditions. Liu et al. (2012) reported upregulation of HSP70 under joint treatment of elevated temperature and *p*CO_2_ in pearl oysters (*Pinctada fucata*).

When larvae were exposed to 1,200 ppm *p*CO_2_ at ambient temperatures, we observed the upregulation of 15 transcripts related to hemocyanin, mannose‐binding proteins, crustin, and c‐type lectin, all of which play roles in pathogen recognition and/or defense (Clark & Greenwood, 2016). These same genes were differentially expressed in greater numbers in the high temperature/high *p*CO_2_ treatment, indicating an overall higher energy input to immune function when both stressors are present. Differential expression of transcripts coding for antilipopolysaccharide factors (ALFs) and their isoforms can indicate individual pathogens through differential expressions (Beale et al., 2008; Clark, Acorn, et al., 2013; Clark, Greenwood,s et al., 2013), but none of these factors were identified as differentially regulated among any of our treatments, indicating that transcripts were nonspecific to known lobster pathogens, and therefore likely a response to treatment conditions. The effect of elevated temperature and *p*CO_2_ on nonspecific immune response could have implications on the *H. americanus* antigen defense systems in future oceanic conditions.

Crustaceans, as a group, have shown relative resistance to end‐century ocean acidification compared to other calcifying organisms (Whitely, 2011). This may be the result of a heightened capacity for ionoregulation, though the energetic trade‐offs. The ramifications of this strategy, particularly with respect to suboptimal food conditions, are still not well understood (Gledhill et al., 2015; Wernberg et al., 2012; Whitely, 2011). For postlarval lobster, we found a clear effect of elevated *p*CO_2_ on gene expression regulation that is enhanced when paired with elevated temperature. These results suggest that crustaceans have molecular mechanisms to respond to these stressors in the postlarval stage. These results complement studies on whole‐organism physiological changes observed in lobster larvae and postlarvae under similar laboratory conditions, and suggest an explanation for why similar studies have found little whole‐organism response to *p*CO_2_ elevated environments. Within early stages, homeostatic compensatory mechanisms could conceal responses in other measured physiological, behavioral, or morphometric end points. However, with added stressors such as low food concentrations, disease, or other immune challenges, the compensatory mechanisms that are apparent with gene expression analysis may exceed metabolic capacity, and manifest in decreased growth, development, or survival.

Understanding the physiological and genetic responses to environmental change is critical to anticipate the effect of warming and acidification on lobster, and the information is needed to improve our ability to predict economic repercussions of climate change on the most valuable single‐species fishery in North America. Characterizing the gene regulatory responses, in particular, can provide a mechanistic understanding of how the vulnerable stages of this species adapt to a rapidly changing environment. Pairing these techniques with whole‐organism physiological and ecological studies will deepen our understanding and ability to anticipate response to environmental changes. As the Gulf of Maine continues to be one of the most rapidly warming coastal areas of the world, the American lobster stands as an icon for the urgency to understand how ocean change is impacting our living marine resources.

This study exposed the larval and postlarval American lobster stages to a future climate scenario during the entirety of their planktonic development, and measured gene regulatory response within its final stage. The design of this study is similar in rearing methodology, treatment conditions, and exposure time to earlier studies that looked at the physiological response of the whole animal (e.g., growth rates, respiration rates, and swimming speed; Small et al., 2015; Waller et al., 2017). The results of those studies generally found little effect of OA relative to responses to temperature. In contrast, the results of this experiment investigated response on gene expression, and found a greater differential expression of genes under OA stress than temperature stress, alone, and an interactive effect when both stressors were present. The results of this experiment provide compelling evidence that compensatory mechanisms at the cellular level minimize the physiological and morphological changes measured in other studies to *p*CO_2_ effects (Small et al., 2015; Waller et al., 2017) and suggests that postlarvae are more responsive to predicted end‐century levels of elevated *p*CO_2_ than previously assumed. The metabolic cost of these compensatory mechanisms is unknown. Experiments using limiting food conditions or sensitive physiological measurements to determine metabolic cost are warranted.

Lacking, though, is a broader understanding of how rapidly populations may be able to adapt to changing conditions. Comparisons of gene regulation among subpopulations along geographic environmental gradients may reveal population‐level differences that could provide new insight into the process of local adaptation. In addition, multigenerational studies would provide a better understanding of the long‐term effects, compensatory ability, and potential for adaptation to warming and acidification.

## CONFLICT OF INTEREST

The authors declare this research was undertaken in absence of any conflict of interest.

## AUTHOR CONTRIBUTION

**Maura Niemisto:** Data curation (lead); Formal analysis (lead); Investigation (equal); Project administration (lead); Visualization (equal); Writing‐original draft (lead). **David M. Fields:** Conceptualization (equal); Funding acquisition (equal); Project administration (equal); Resources (equal); Supervision (equal); Writing‐review & editing (equal). **K. Fraser Clark:** Data curation (equal); Formal analysis (equal); Methodology (equal); Resources (equal); Software (equal); Supervision (equal); Writing‐review & editing (equal). **Jesica D. Waller:** Data curation (equal); Investigation (equal); Methodology (equal); Project administration (equal); Writing‐review & editing (equal). **Spencer J. Greenwood:** Conceptualization (equal); Funding acquisition (equal); Methodology (equal); Project administration (equal); Resources (equal); Supervision (equal); Writing‐review & editing (equal). **Richard A. Wahle:** Conceptualization (equal); Funding acquisition (equal); Methodology (equal); Project administration (equal); Resources (equal); Supervision (equal); Writing‐review & editing (equal).

## Data Availability

Data sequences are available at www.ncbi.nlm.nih.gov/bioproject/PRJNA669582. Accession: PRJNA669582 ID: 669582.
